# Assessment of chromatin remodeling of acute myeloid leukemia cells treated with gilteritinib: a case report

**DOI:** 10.1186/s13256-025-05186-2

**Published:** 2025-04-03

**Authors:** Jinichi Mori, Takahiro Sawada, Koki Nojiri, Yoshiaki Kanemoto, Tadashi Okada, Tomohiro Kurokawa, Shigeaki Kato

**Affiliations:** 1 Research Institute of Innovative Medicine, Tokiwa Foundation, Iwaki, Fukushima 972-8322 Japan; 2 Graduate School of Life Science and Engineering, Iryo Sosei University, 5-5-1, Iino, Chuo-Dai, Iwaki, Fukushima 970-8551 Japan; 3 Medical Epigenome Research Laboratory, School of Medicine, School of Graduate Education, Fukushima Medical University, 1 Hikarigaoka, Fukushima, 960-1295 Japan; 4 Department of Hematology, Jyoban Hospital, Tokiwa Foundation, Iwaki, Fukushima 972-8322 Japan

**Keywords:** Gilteritinib, Epigenetics, Drug resistance

## Abstract

**Background:**

Acute myeloid leukemia is a hematological malignancy characterized by acquired genomic aberrations. Mutations in the *FMS-like tyrosine kinase 3* gene cause constitutive activation of downstream signaling pathways, thereby driving disease progression and conferring a poor prognosis. Gilteritinib, a tyrosine kinase inhibitor, is a promising treatment for *FMS-like tyrosine kinase 3*-mutated acute myeloid leukemia. However, gilteritinib resistance remains a significant concern, and its underlying mechanisms are not yet understood.

**Case presentation:**

A 65-year-old Japanese male patient who was receiving regular hemodialysis developed pancytopenia in 2017. He required recurrent red blood cell transfusions due to anemia in 2018. In 2019, he was diagnosed with myelodysplastic syndrome with excess blasts. We administered three courses of azacitidine but ceased it due to severe cytopenia. His disease had transformed to acute myeloid leukemia. The fourth course of azacitidine was administered, but it was ineffective. Since a tyrosine kinase domain mutation in *FMS-like tyrosine kinase 3* was detected in the acute myeloid leukemia cells, we administered gilteritinib at a dose of 120 mg. Although the treatment initially showed efficacy, the disease progressed, and he died 7 days after the initiation of gilteritinib. To assess the epigenetic changes in acute myeloid leukemia during the treatment with gilteritinb, we performed the assay for transposase-accessible chromatin with sequencing using the leukemia cells obtained from the patient before and after gilteritinib treatment. After the treatment, greater than fivefold changed assay for transposase-accessible chromatin peaks were detected in 137 (upregulated) and 105 (downregulated) regions. Among them, half of the regions were located in the intergenic regions. A Gene Ontology analysis of affected genes listed the mitogen‑activated protein kinase pathway, which is potentiated by the *FMS-like tyrosine kinase 3* genetic mutations in leukemia cells. No significant changes were noted at the *FMS-like tyrosine kinase 3* locus. On the gene locus of *PPP2R2B,* a known cancer-associated gene, the peaks were decreased, suggesting reduced chromatin accessibility. Conversely, upregulation peaks were observed on the gene locus and adjacent noncoding region of *PDGFD* that is associated with the progression of various types of cancer including acute myeloid leukemia.

**Conclusions:**

Our study demonstrated the epigenetic changes in acute myeloid leukemia cells that may be associated with gilteritinib resistance.

**Supplementary Information:**

The online version contains supplementary material available at 10.1186/s13256-025-05186-2.

## Background

Acute myeloid leukemia (AML) is a heterogeneous disease caused by various cytogenetic abnormalities and genetic mutations [1, 2]. Constitutive and ligand-independent activation of *FMS-like tyrosine kinase 3* (*FLT3*) by genetic mutations is considered a driver of AML. The *FLT3* mutations are found in approximately 30% of AML cases. Internal tandem duplication and point mutations in the tyrosine kinase domain (TKD) result in a gain of membrane receptor functions of *FLT3* in downstream intracellular signaling [3]. Patients with AML acquiring such genetic mutations exhibit poor prognosis presumably due to enhanced cell proliferation and prolonged survival of leukemia cells [4]. Gilteritinib, a second-generation type I tyrosine kinase inhibitor of FLT3, has been developed for AML harboring the *FLT3* mutations. It also serves as an effective inhibitor for the other receptor tyrosine kinase, AXL, whose hyper-expression in the AML cells leads to prolonged leukemia cell survival along with chemoresistance [5]. The ADMIRAL study found that in patients with relapsed or refractory AML harboring the *FLT3* mutations, treatment with gilteritinib significantly prolonged overall survival as compared with other chemotherapies [6]. However, the complete remission rate with gilteritinib was only 21%, and relapse was inevitable even among patients who initially responded. Therefore, elucidating the molecular mechanisms that confer resistance to gilteritinib is critical for overcoming this therapeutic challenge. By detailed analysis, gilteritinib was found to be more beneficial for the patient subgroup carrying genetic mutations for the enzyme DNA methyltransferase 3A (*DNMT3A*) [7]. DNMT3A is pivotal in epigenetic regulation through DNA methylation leading to chromatin silencing and its inhibitor azacitidine is used for patients with myelodysplastic syndrome and AML [8, 9]. Therefore, in AML cells with *FLT3* mutations, dysregulation of the epigenetic programme might be possible. To test this hypothesis, we examined genome-wide chromatin accessibility using assay for transposase-accessible chromatin with sequencing (ATAC-seq) to determine whether gilteritinib can remodel the chromatin landscape in leukemia cells of an AML case with mutations in the *FLT3* TKD.

## Case presentation

### Patient details

**Fig. 1:**
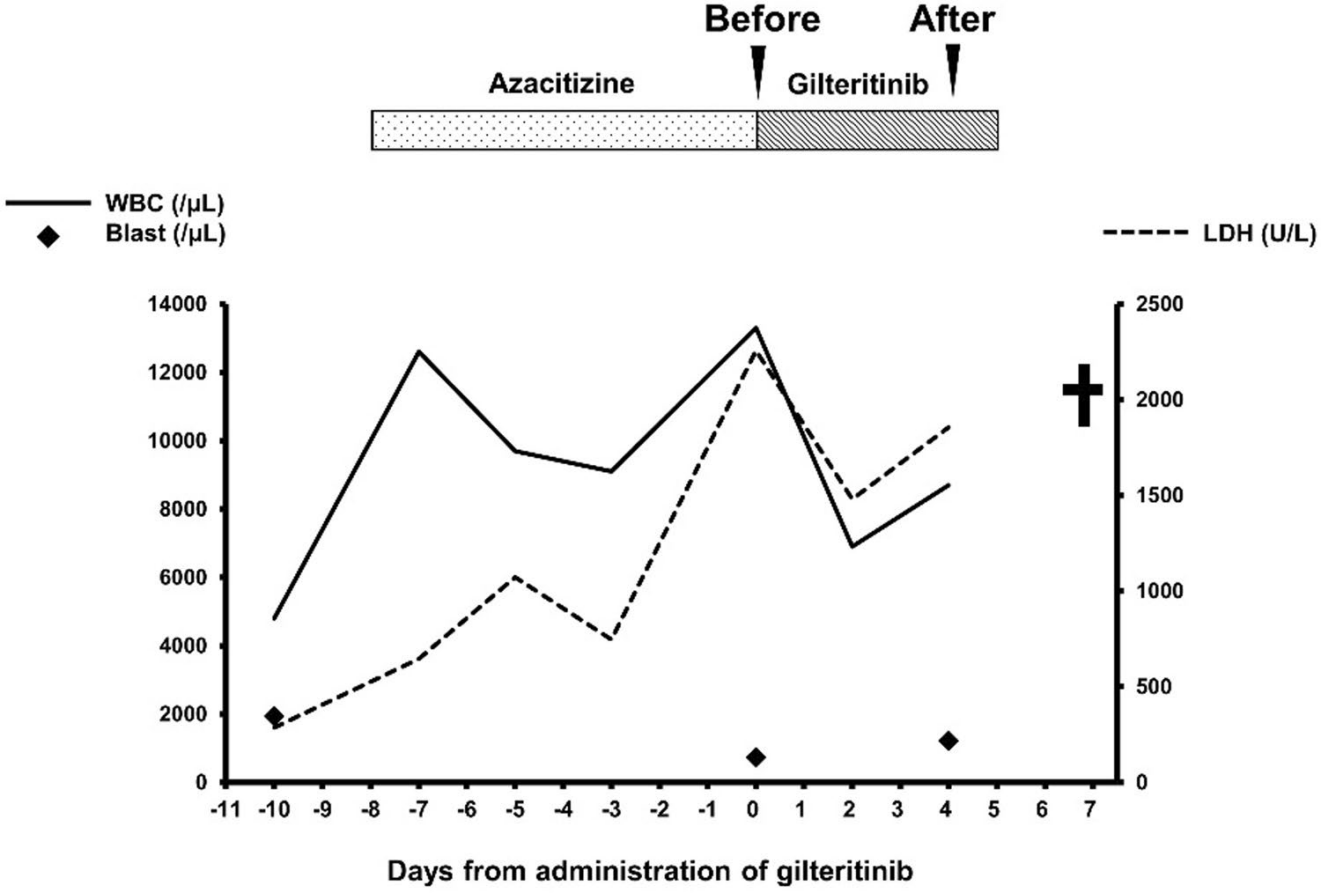
Clinical course of the patient during treatment with azacitidine and gilteritinib. Black arrows indicate the timings of blood sampling and black cross represents the death of the patient

A 65-year-old Japanese man who had been undergoing regular hemodialysis for end-stage renal disease of unknown etiology since his 30s developed pancytopenia in 2017. His anemia made him dependent on regular red cell transfusion in 2018. In February 2019, we performed a bone marrow examination and found dysplasia in granulocytes and erythroid lineages with 1.8% of myeloblasts. G-banding method revealed normal karyotype. We thus diagnosed myelodysplastic syndrome with excess blasts in the recent International Consensus Classification [10] and myelodysplastic neoplasm increased blasts 1 in the 5th edition of WHO classification [11]. We administered three courses of azacitidine but ceased it due to severe cytopenia. The blast cells increased in his peripheral blood with disseminated intravascular coagulation in November 2019, indicating transformation to AML. To reduce the blast cells, the fourth course of azacitidine was administered, but it was ineffective. Since the genetic analysis identified a mutation in the tyrosine kinase domain of *FLT3* in the AML cells, we started to administer 120 mg of gilteritinib every day (Fig. 1). Although gilteritinib showed a temporary effect of decreasing white blood cells and lactate dehydrogenase on day 2 after initiation without obvious adverse events, they began to rise on day 4, and the treatment was deemed ineffective. We decided to terminate treatment for AML as well as hemodialysis and to use the best supportive care. The patient subsequently died of arrhythmia on day 7.

Written consent for genome sequencing and publication had been obtained from the patient. This research was approved by the local institutional review board (approval no. JHTF-2019-003) and conducted ethically following the World Medical Association Declaration of Helsinki.

### Sample collection and processing

Blood samples were collected immediately before the first dose of gilteritinib (referred to as “before” in this study) and 4 days after the initiation of the treatment (referred to as “after” in this study) (Fig. 1). The CD34^+^ cells were isolated from the blood samples using CD34 MicroBead Kit-UltraPure (Miltenyi Biotec, Gladbach, Germany) following the manufacturer’s instructions. CD34^+^ cells were quantified using a flow cytometer at Kotobiken Medical Laboratory (Ibaraki, Japan).

### Assay for transposase-accessible chromatin (ATAC) with high-throughput sequencing

Library preparation, sequencing, mapping, and Gene Ontology (GO) analysis for ATAC-seq were performed at DNAFORM (Yokohama, Japan). Cells were lysed, and the rearrangement reaction was performed using Tn5 transposase (#FC121-1030, Illumina CA, USA) for 30 minutes at 37 ℃. The reaction solution was purified using the MinElute PCR Purification Kit (#28004, Qiagen, Hilden, Germany). Next, five cycles of polymerase chain reaction (PCR) using custom Nextera PCR primers were conducted using NEBNext Q5 Hot Start HiFi PCR Master Mix (#M0543, New England Biolabs, MA, USA). The number of additional PCR cycles was determined using qPCR of the partially amplified product. PCR products were prepared using Agencourt AMPure XP beads (#A63881, Beckman Coulter, CA, USA) following the manufacturer’s instructions. Paired-end sequencing was performed on the NextSeq 500 instrument (Illumina). The obtained reads (GEO accession number GSE210617) were mapped on the basis of the human GRCh38.p13 genome using the Burrows–Wheeler aligner (BWA-MEM: ver. 0.7.17-r1188).

### Bioinformatic analysis of ATAC-seq

Bioinformatic analysis was performed as described previously [12]. Peak calling was performed using MACS2 (ver. 2.2.6) and epic2 (ver. 0.0.41) with default parameters. Peak annotation was performed using HOMER (ver. 4.9.1). For GO analysis, we first extracted peaks that exhibited at least a twofold increase in ATAC signal before and after gilteritinib treatment. We then compiled a list of the protein-coding genes nearest to each peak and performed the Kyoto Encyclopedia of Genes and Genomes (KEGG) pathway analyses using DAVID2021 (https://david.ncifcrf.gov/tools.jsp). The differential peaks were identified by calculating the log2FC value (< −0.75 or > 0.75). Next, the functions and transcription factor regulations among the peaks were analyzed by comparison with the transcription active site (Histone H3K27 acetylation site) or transcriptional repression site (Histone H3K9 di-methylation site) using ChIP-Atlas (http://chip-atlas.org/) [13]. Histone acetylation or methylation site data in the leukemia cells of the patient with AML were obtained using Chromatin Immunoprecipitation (ChIP) of H3K27ac (SRX4496694) and H3K9me2 (SRX1136016), respectively. The motif enrichment analyses were performed using the MEME-ChIP algorithm [14].

## Results

**Fig. 2:**
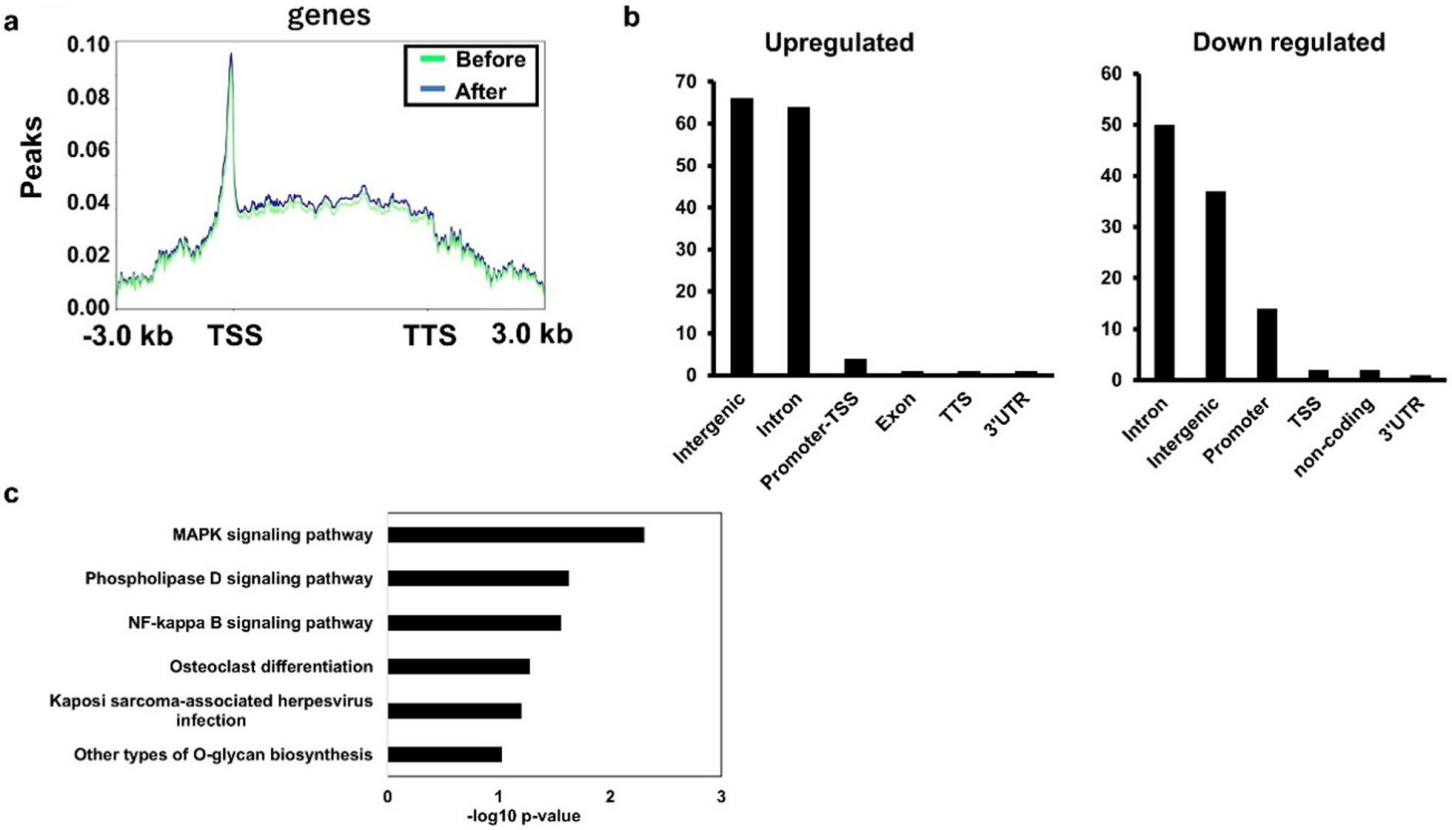
**a** Mapping profile of assay for transposase-accessible chromatin (ATAC) sequencing data (GSE210617) analyzed using deepTools. All genes with one or more reads were mapped by averaging the read depth at each position from 3 kb upstream of the transcription start site (TSS) to 3 kb downstream of the transcription termination site (TTS) and normalizing the ATAC signals. **b** Genomic location annotation analysis of ATAC-seq peaks of after samples upregulation relative to the before samples. *UTR* untranslated region. **c** KEGG pathway analysis of the genes in the after samples. The gene names on the right represent the genes enriched in each term, and the horizontal bars indicate their corresponding *p*-values. The asterisks denote statistical significance (*p* < 0.05)

**Fig. 3:**
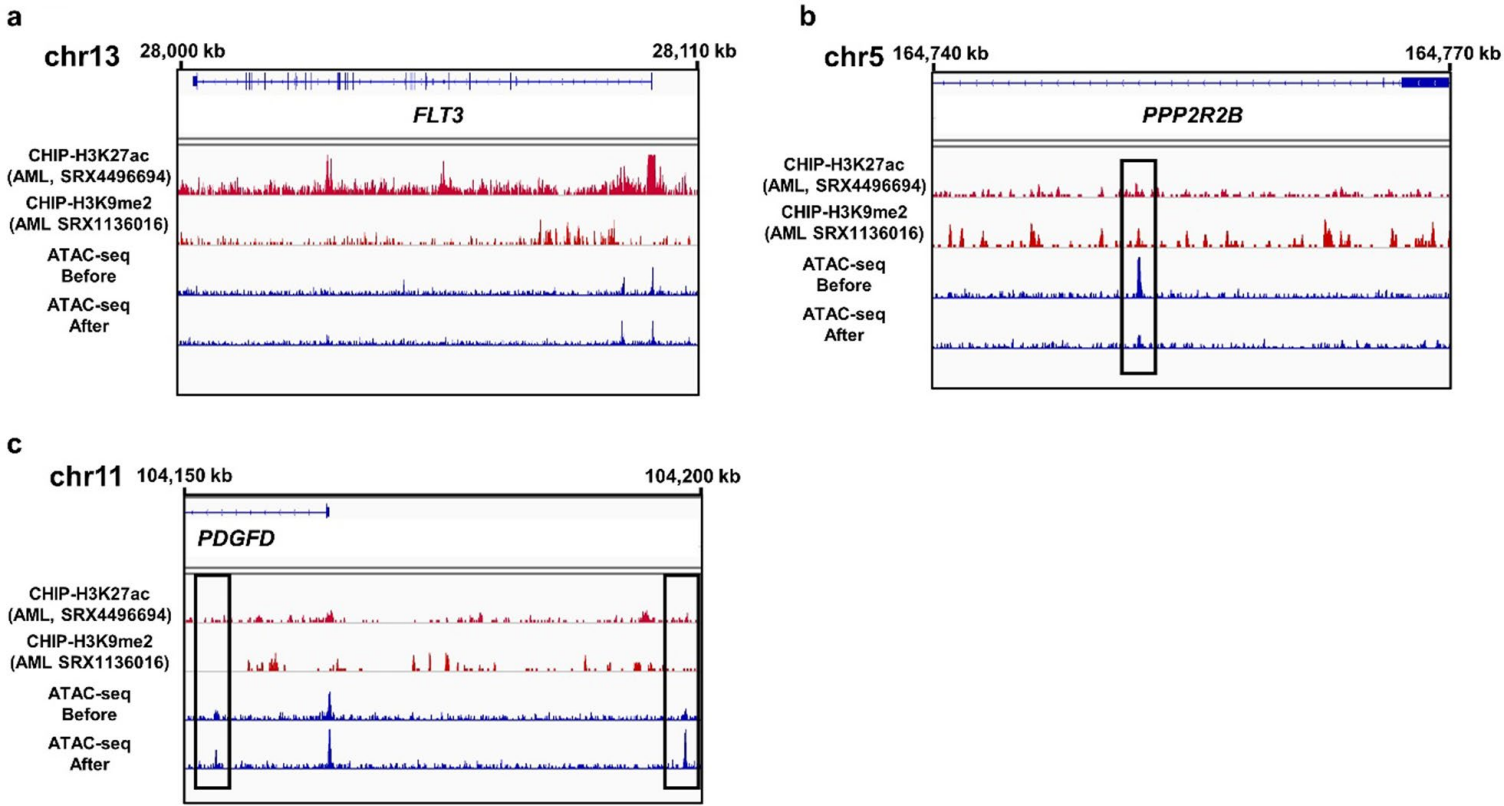
**a**–**c** Integrated genomics viewer tracks of ATAC-seq data (bottom) and ChIP-atlas data in acute myeloid leukemia by ChIP of H3K27ac (SRX4496694) and H3K9me2 (SRX1136016) or CTCF (SRX188951) for each gene locus on chr13, chr 5, and chr 11, respectively. The peak score is 0.0–0.5

The laboratory data of the patient at before and after timepoints are presented in Table 1. Immature granulocytes along with blast cells seen in the peripheral blood before the treatment, remained until day 4 (after). From the before and after specimens, 4.66 × 10^3^ and 2.14 × 10^3^ of CD34^+^ cells were isolated, respectively (Additional file 1: Fig. S1). They were subsequently analyzed using ATAC-seq. As expected, the signal peaks were concentrated around transcription start sites (TSS) (Fig. 2a). After the treatment, greater than fivefold changed peaks were detected in 137 (upregulated) and 105 (downregulated) regions (Fig. 2b). Along with the affected regions, half of the regions (66 out of 137 upregulated and 37 out of 105 downregulated) were located in the intergenic regions that were assumed to serve as regulators for the adjacent genes. A GO analysis of affected genes listed the mitogen‑activated protein kinase pathway, consistent with the previous findings of intracellular signalling pathways potentiated by the *FLT3* genetic mutations in leukemia cells [15]. We then took a closer look at the loci of the listed genes associated with cancers. No alteration on the *FLT3* gene locus was seen due to the treatment (Fig. 3a). On the gene locus of *PPP2R2B* (a subunit of tumor suppressor protein phosphatase 2A) [16], the peaks were decreased, suggesting reduced chromatin accessibility (Fig. 3b). In contrast, upregulation peaks were observed on the gene locus and adjacent noncoding region of platelet-derived growth factor D (*PDGFD*) that is associated with the progression of various types of cancer, including AML (Fig. 3c) [17]. As the profiles of chromatin openness were affected, we tested whether de novo binding of DNA-binding transcription factors was generated by gilteritinib. By a motif enrichment analysis of DNA binding sites of transcription regulators in the open chromatin areas, no significant alteration of enrichment was seen between before and after treatment scenarios (Additional file 1: Fig. S2).

**Table 1:** Laboratory data before and after administration of gilteritinib

Before (day 0)		After (day 4)	
WBC	**13,300/µL**	WBC	**8700/µL**
baso	0.0%	baso	0.0%
mono	4.0%	mono	6.5%
eosino	0.5%	eosino	0.0%
Lymph	17.0%	Lymph	20.0%
stab	10.5%	stab	4.5%
seg	23.5%	seg	26.0%
meta	5.0%	meta	2.5%
myelo	33.5%	myelo	26.0%
promyelo	0.5%	promyelo	0.5%
blast	5.5%	blast	14.0%
RBC	**225 × 10**^**4**^** /µL**	RBC	**219 × 10**^**4**^** /µL**
Hb	6.8 g/dL	Hb	6.5 g/dL
Hct	20.1%	Hct	19.6%
Ret	1‰	Ret	1‰
MCV	89	MCV	90
MCH	30.2	MCH	29.7
MCHC	33.8	MCHC	33.2
Plt	0.6** × **10^4^ /µL	Plt	0.6** × **10^4^ /µL
Alb	3.1 g/dL	Alb	3.1 g/dL
BUN	90.4 mg/dL	BUN	97.2 mg/dL
Cr	5.10 mg/dL	Cr	4.56 mg/dL
AST	40 U/L	AST	47 U/L
ALT	6 U/L	ALT	9 U/L
LDH	2255 U/L	LDH	1855 U/L
ALP	315 U/L	ALP	317 U/L
Na	129 mEq/L	Na	131 mEq/L
K	5.4 mEq/L	K	5.0 mEq/L
CRP	8.99 mg/L	CRP	3.12 mg/L

*ALT* alanine aminotransferase, *AST* aspartate aminotransferase, *Alb* albumin, *ALP* alkaline phosphatase, *BUN* blood urea nitrogen, *Cr* creatinine, *CRP* C-reactive protein, *Hb* hemoglobin, *Hct* hematocrit, *K* potassium, *LDH* lactate dehydrogenase, *MCH* mean corpuscular hemoglobin, *MCHC* mean corpuscular hemoglobin concentration, *MCV* mean corpuscular volume, *Na* natrium, *Plt* platelets, *RBC* red blood cells, *WBC* white blood cells

## Discussion

In this study, we assessed the effects of gilteritinib on chromatin openness in a patient with AML encompassing a known gain of the function point mutation in the *FLT3* TKD. As this patient was pretreated with a DNMT3A inhibitor (azacitidine) and as gilteritinib is effective for patients with relapsed or refractory AML with *FLT3* genetic mutations, we presumed that gilteritinib is potent enough to remodel chromatin landscape from an abnormal into a normal state. However, this drug was found to be ineffective for the present patient. We could detect an alteration in the chromatin landscape when chromatin openings were assessed by ATAC-seq in the leukemia cells isolated from the blood samples. Since the past findings suggested that gilteritinib ameliorates hyper-activated intracellular signaling-mediated STAT5A, RAS, MEK, and PI3K/AKT by silencing the tyrosine kinase activity of mutated *FLT3* [18], we presumed that the disturbed intracellular signalling in leukemia cells of the patient was improved, at least in part, as the initial dose appeared partially effective to attenuate the disease progression. In our case, where the disease progressed even after administration of gilteritinib, we observed the epigenetic features associated with drug resistance rather than the drug’s effectiveness. For example, *PPP2R2B*, which is a subunit of tumor-suppressor protein phosphatase 2A, was listed in the “downregulated gene” [16]. In contrast, *PDGFD*, an oncogenic gene [14], was upregulated in our case. We found multiple binding sites of BRD4, a BET family protein, in ChIP-atlas database (data not shown), suggesting that the expression levels of these genes are regulated by BRD4. An in vitro study demonstrated that combination of gilteritinib and a BET inhibitor synergistically induced apoptosis in *FLT3*-mutated AML cells [19]. These findings suggest that the addition of a BET inhibitor to gilteritinib may resolve the BRD4-mediated dysregulation of oncogenes or tumor suppressor genes associated with gilteritinib resistance, thereby enhancing the drug’s sensitivity.

For patients in whom it is effective, the gene expression profile, as well as epigenetic modulation, may be benefited from gilteritinib treatment. The precise action of gilteritinib for AML cells with *FLT3* genetic mutations in chromatin remodeling should be studied further when drug-sensitive and insensitive leukemia cells were compared with more comprehensive whole genome analyses such as RNA sequencing and chromatin immunoprecipitation (ChIP) sequencing.

## Conclusion

We explored the change of chromatin openness in AML cells before and after the administration of gilteritinib treatment. In AML cells of the gilteritinib-resistant case, approximately half of the affected regions were located in the intergenic regions, which possibly reflected changes related to drug resistance. Since gilteritinib resistance may arise not only from somatic mutations in protein coding genes, but also from complex regulatory abnormalities in noncoding regions, a comprehensive multimodality analysis is essential to elucidate the underlying mechanism.

## Supplementary Information

**Additional file 1: Fig. S1**. Flow-cytometric results for CD34 + cells isolated from the blood samples before (left) and after (right) the treatment with gilteritinib. The fluorescence intensities of 7-amino-actinomycin D (7-AAD) (*y*-axis) and CD34 (*x*-axis) are shown in the dot plots. The gated region represents CD34 + and 7-AAD- leukemia blasts. **Fig. S2.** Motif enrichment analysis of AML cells before and after treatment. DNA-binding motifs included in the opened chromatin regions in AML cells before and after treatment with gilteritinib are shown. Listed are transcription factors known to bind the motifs.

## Data Availability

Although consent for information disclosure has been obtained from the patient, it contains sensitive personal information. Therefore, raw data will be provided upon request by contacting the corresponding author.

## Abbreviations

AML: Acute myeloid leukemia
TKD: Tyrosine kinase domain
ATAC-seq: Assay for transposase-accessible chromatin with sequencing
PCR: Polymerase chain reaction
qPCR: Quantitative polymerase chain reaction
ChIP: Chromatin immunoprecipitation
