# Supplementary figures and images for: Assessment of chromatin remodeling of acute myeloid leukemia cells treated with gilteritinib: a case report
Source: J Med Case Rep. 2025 Apr 3;19:153. doi: 10.1186/s13256-025-05186-2 (PMC11966927; doi:10.1186/s13256-025-05186-2)

Fig. S1

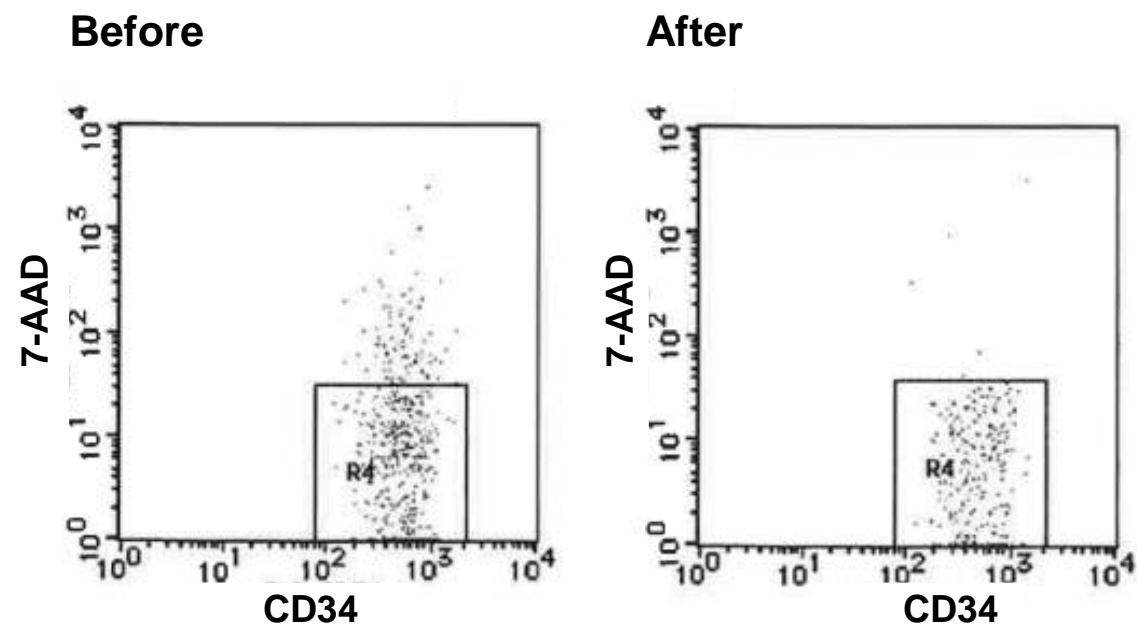

**Fig. S2**

## Before

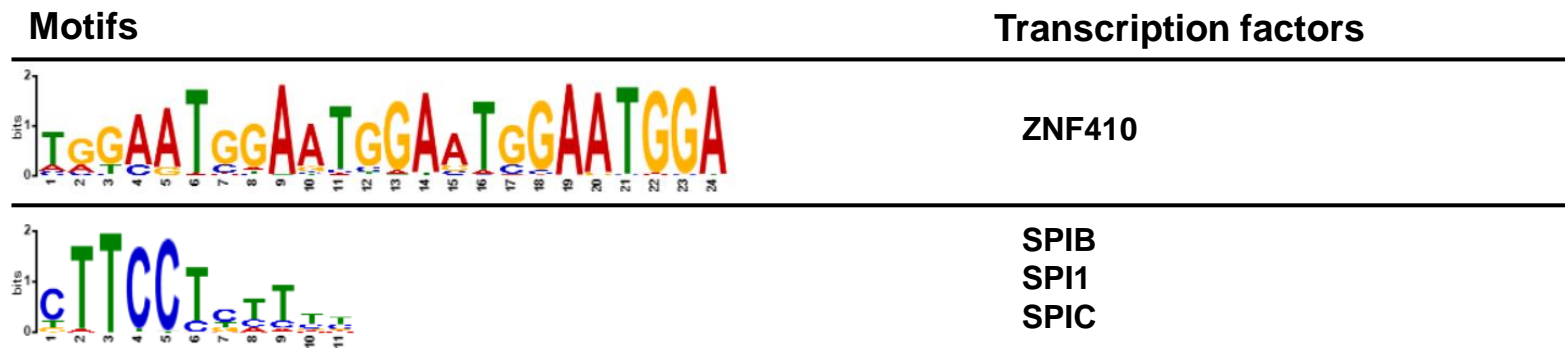

## After

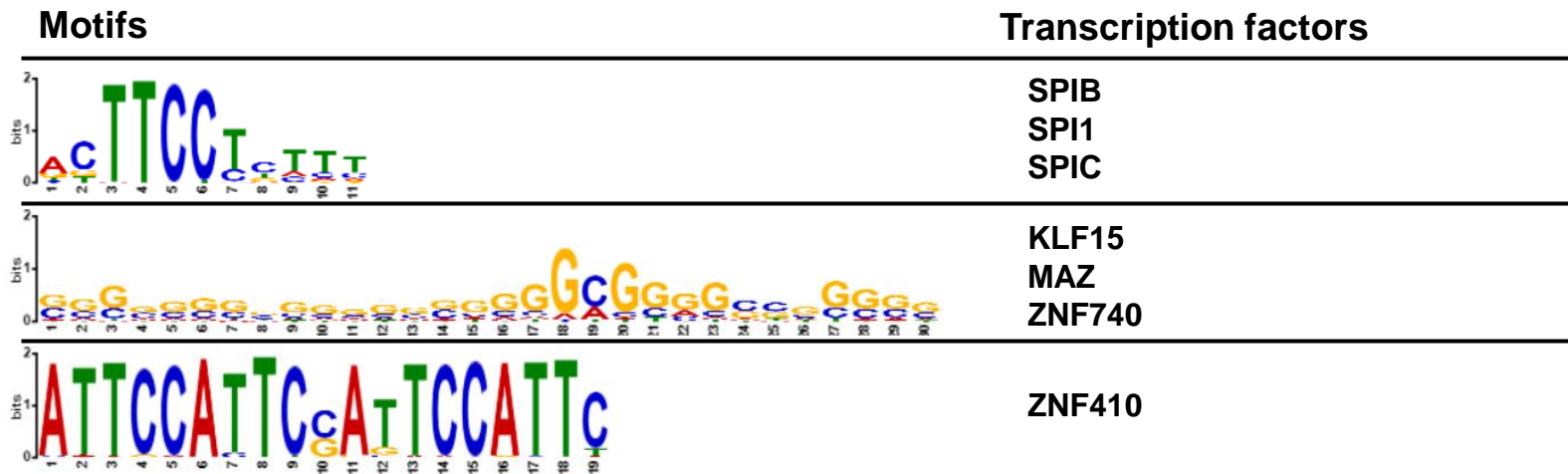

Supplement: Supplementary file 1 — Additional file 1: Fig. S1. Flow-cytometric results for CD34 + cells isolated from the blood samples before (left) and after (right) the treatment with gilteritinib. The fluorescence intensities of 7-amino-actinomycin D (7-AAD) (y-axis) and CD34 (x-axis) are shown in the dot plots. The gated region represents CD34 + and 7-AAD- leukemia blasts. Fig. S2. Motif enrichment analysis of AML cells before and after treatment. DNA-binding motifs included in the opened chromatin regions in AML cells before and after treatment with gilteritinib are shown. Listed are transcription factors known to bind the motifs. [file 13256_2025_5186_MOESM1_ESM.pdf]
